# Exosome-orchestrated hypoxic tumor microenvironment

**DOI:** 10.1186/s12943-019-0982-6

**Published:** 2019-03-30

**Authors:** Wanrong Meng, Yaying Hao, Chuanshi He, Ling Li, Guiquan Zhu

**Affiliations:** Department of Head and Neck Oncology, Sichuan Cancer Hospital & Institute, Sichuan Cancer Center, School of Medicine, University of Electronic Science and Technology of China, No. 55, Section 4, Renmin South Road, 610041 Chengdu, Sichuan People’s Republic of China

**Keywords:** Hypoxia, Tumor microenvironment, Exosome, Extracellular vesicle, Non-coding RNA, miRNA

## Abstract

Hypoxic tumor microenvironment is a common feature of solid tumors and is associated with aggressiveness and poor patient outcomes. A continuous interference between cancer cells and stromal cells within the hypoxic microenvironment has been uncovered for its importance in cancer development and treatment responsiveness. Exosomes, initially considered as “garbage bins” for unwanted material from cells, are now elucidated to perform a variety of functions that involve interactions within the cellular microenvironment due to their ability to carry numerous cargoes, including lipids, proteins, nucleic acids, and metabolites. Exosome-mediated continuous interference between cancer cells and stroma are believed to regulate hypoxia-adaptation and to rebuild the microenvironment in return. In this review, we will discuss the knowledge in literature with respect to the exosome-mediated multi-directional and mutual signal transmission among the variety of cell types within hypoxic cancer microenvironment.

## Background

The “seed and soil theory” described by Stephen Paget [1] in the 1880s, for the first time, implied the role of tumor microenvironment (TME) in the initiation and maintenance of tumorigenesis. The TME is an internal physical and chemical condition that cancer cells live in, which is dynamically composed by extracellular matrix (ECM), blood vessels, stromal cells (e.g. immune cells, fibroblasts, endothelial cells, and mesenchymal stem cells), and secreted factors such as cytokines and growth factors [2]. One of the most intensively studied characteristics of TME is hypoxia, defined as a reduction in the normal level of tissue oxygen tension [3, 4]. The hypoxic TME is involved in many “hallmarks of cancer” [5], such as angiogenesis [6], reprogramming energy metabolism [7], evading immune destruction [8], activating invasion and metastasis [9], tumor-promoting inflammation [10], sustaining proliferative signaling [3], resisting cell death [3], and genome instability [7]. As such, hypoxic TME has gained much scientific attention in the past decades. Nowadays, there is growing body of new findings to improve the understanding of hypoxia-regulated cancer biology, one of which is the exosome-mediated communications within TME. In this review, we will discuss the knowledge in literature with respect to the exosome-mediated multi-directional and mutual signal transmission among the variety of cell types within hypoxic cancer microenvironment.

### Exosomes

In the past decade, a booming interest has been paid to exosomes in the cancer research, mainly due to the discovery of functional molecular cargos in exosomes that allow them to operate as signaling platforms for information delivery between cells [11].

Exosomes are defined as a class of extracellular vesicles (EVs) formed by inward budding of endosomal membrane and releasing into the extracellular environment upon fusion with the plasma membrane [12, 13]. Early in 1960s, exosomes (formerly called ‘platelet dust’) were initially described as subcellular structure originated from normal platelets [14]. The term “exosome” was first described in reticulocytes during the maturation of erythrocytes by Rose M. Johnstone et al. in 1980s [15]. Exosomes have been long-term silenced for their presumed role as cellular “garbage dumpsters”. This is about to change in 1996, since exosomes derived from B lymphocytes was found to induce antigen-specific MHC-II-restricted T cell responses, suggesting an active function by exosomes in antigen presentation [16]. Thereafter, an encouraging progress in exosome research has been obtained on many aspects of exosome biology, such as biogenesis and release, morphology, contents, isolation technique, and functions, especially.

### Exosome morphology and size

It has been well documented that exosomes usually appear as cup-shaped under transmission electron microscopy, with a density between 1.13 and 1.17 g/ml, and expressing CD63, Alix, VPS35, galectin 3, HSP90, fibronectin, and placental alkaline phosphatase [17]. The size of exosomes, however, remains inconclusive, with varied descriptions of 20–100 nm [18], 30–100 nm [12, 19], 40–100 nm [20], 30–150 nm [21], 40–150 nm [11], and 50–100 nm [17] in different review papers.

We suggest that 30-100 nm in diameter is the most acceptable description of exosome for several reasons. Firstly, after the initial description of exosomes in reticulocytes with 30–50 nm of diameter [22], exosomes were then found to be 60–80 nm from B lymphocytes [16], 60–90 nm from DCs [23], 40–100 nm from platelets [24], 30–90 nm from intestinal cells [25], and 60–90 nm from human and mouse tumor cells [26]. With enough respect to the discovery history of exosomes, 30–100 nm of diameter covers the range of exosome size derived from different cell types. Secondly, vesicles less than 30 nm in diameter are too small to be observed by photon microscopy [19]. And circulating particles > 100 nm in size are vulnerable to clearance by the mononuclear phagocyte system [27]. Finally, vesicles > 100 nm in size represent the morphology of microvesicles that are formed by shedding from cell surface [24]. Therefore a diameter of 30–100 nm represents a typical range of exosome size in various cell types.

### Exosome contents

In the past decade, studies have revealed that exosomes can carry numerous cargoes, including lipids, proteins, nucleic acids, and metabolites. Exosomal cargos are dependent on the parent cell type and vary between different physiological or pathological conditions in which the donor cells live. Several databases (i.e. ExoCarta [28], EVpedia [29], and Vesiclepedia [30]) have been built up to provide information about exosomal cargos, hosting > 1000 lipid, > 40,000 proteins, and > 7000 RNAs entries cataloged from 10 different species. Given the large amount of cargos being delivered by exosomes, only a small set of them has their functions revealed in cancer biology, emphasizing the necessity of further investigation.

### Hypoxia regulate exosome production

We and several other groups have provided direct evidence demonstrating an increased production of exosomes in response to hypoxia [31–34] and hypoxia-related conditions such as low pH [35] and oxidative stress [36]. King et al. [33] exposed breast cancer cell lines to moderate (1% O_2_) and severe (0.1% O_2_) hypoxia and found a significant increase in exosome number in a hypoxia-inducible factor (HIF)-1α-dependent manner. Wang et al. [32] further demonstrated that HIF-1α induces exosome release through transactivating the small GTPase RAB22A, which colocalizes with budding vesicles at the surface of breast cancer cells. We showed that oral squamous cell carcinoma (OSCC) cells secreted increased amount of exosomes under hypoxic condition, in which process, HIF-1α and HIF-2α played overlapping roles [31]. In keratinocytes, the proline-rich Akt substrate of 40 kDa (PRAS40) was supposed to be required for hypoxia-induced exosome secretion [34].

Other than direct evidences listed above, there are several clues to support the machineries involved in the hypoxia-regulated exosome biogenesis. Firstly, RHO-associated protein kinase (ROCK), an important regulator of actin dynamics, can induce exosome biogenesis in different types of tumor cells [37]. The expression of ROCK [38] has been confirmed to be induced by hypoxia. Secondly, activation of calpain can cause the shedding of micro vesicles from the membrane of aggregating platelets [39]. The mechanism by which calpain regulate exosome biogenesis might be that it drives rearrangements in the asymmetry of membrane phospholipids, which causes physical bending of the membrane and restructuring of the underlying actin cytoskeleton, favoring membrane budding and formation of exosomes [12]. Hypoxia has been suggested to increase mRNA and protein amount of calpain and elicit calpain activation in macrophages [40]. These direct and indirect evidences suggest that exosome production is up-regulated under hypoxic microenvironment, albeit the mechanisms by which still need further clarification.

### Hypoxic exosomal biomarkers

Hypoxia substantially alters the proteomic and nuclear acid profiles of exosomes [31, 41], making them a potential noninvasive biomarker (probe) for hypoxic status of tumors. Studies performed by Kucharzewska et al. [42] with patient materials revealed enrichment in exosomes of hypoxia-regulated mRNAs and proteins (e.g., MMPs, IL-8, PDGFs, caveolin 1, and lysyl oxidase), several of which were associated with poor glioma patient prognosis. They concluded that the proteome and mRNA profiles of exosomes closely reflect the oxygenation status of donor glioma cells and patient tumors and that the exosomal pathway constitutes a potentially targetable driver of hypoxia-dependent intercellular signaling during tumor development [42]. In addition, HIF-1α itself was found in exosomes with transcriptional activity which has been widely accepted as a good biomarker to predict cancer progression as well as treatment outcomes [43]. Consistently, we showed that circulating exosomal miR-21, one of the most significantly upregulated miRNAs under hypoxia, was closely associated with hypoxic status in patients with OSCC [31]. Very recently, an exosome-based platform has been developed to monitor the tumor hypoxia in vivo using magnetic particle imaging [44]. It was showed that hypoxic cells preferentially take up exosomes released by hypoxic donor cells, demonstrating the ability of hypoxic cell-derived exosome as a hypoxia detection probe [44]. Furthermore, this hypoxic exosome-based platform was also efficient in delivering anti-cancer drug and radiosensitizers to various kinds of hypoxic cancers and imaging the efficacy of treatment using magnetic particle imaging [44].

Since hypoxia and expression of hypoxia-related biomarker as associated with cancer progression and treatment responsiveness, various strategies, such as oxygen electrodes, chemical probes, immunohistochemistry, and molecular imaging, have been adopted to assess tumor hypoxia in various cancer types [41]. The exosomal biomarker represents a novel and noninvasive strategy for hypoxia measurement in vivo and for appropriate clinical decision making. However, the identification of tumor specific exosomes in the peripheral blood remains challenging, since they contain mainly overlapping surface markers as non-tumor-derived exosomes.

### Tumor derived exosome (TDEs) in hypoxic microenvironment

**Fig. 1:**
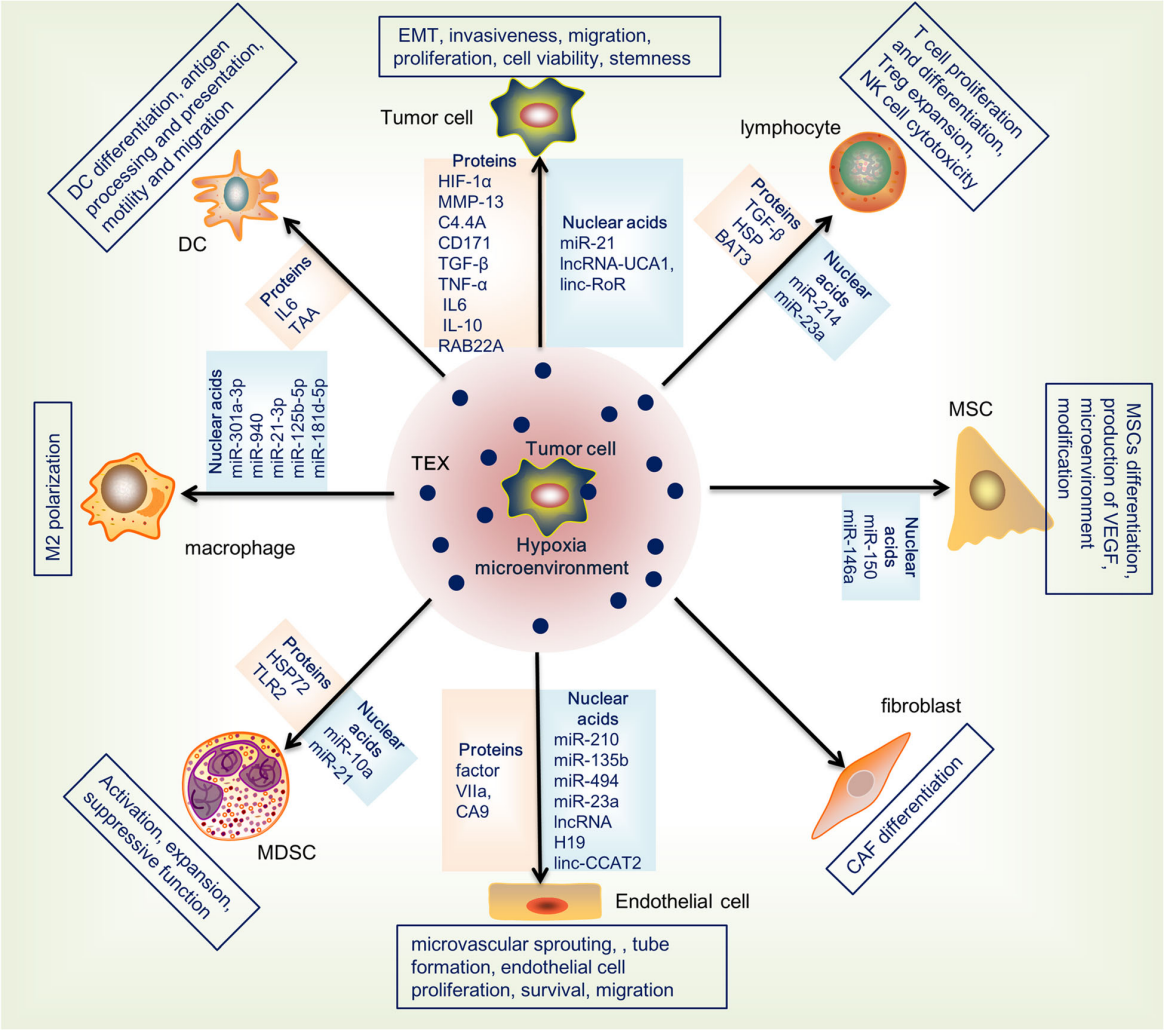
A schematic representation of hypoxic TDEs in regulating tumor cells and a variety of stromal cells

The tumor-derived exosomes (TDEs) are enriched in the TME, delivering tumoral signaling to both tumor cells and stromal cells and playing fundamental functions in a wide array of pathological scenarios, such as tumor invasiveness, angiogenesis, proliferation, chemotherapy and radiation resistance, immune evasion, metabolism, and cancer stemness (Fig. 1).

### TDE-mediated crosstalk between cancer cells

The TDE-mediated tumor-tumor cell communications within hypoxic microenvironment have been extensively investigated for their roles in regulating cancer invasiveness and metastasis in nasopharyngeal [43, 45], oral [31], bladder [46], lung [47], prostate [48], breast [32], pancreatic [49], and ovarian [50] carcinomas through carrying a variety of proteins and nuclear acids.

### Proteins

Proteins represent one of the most important exosomal cargos that involved in hypoxia regulation. Aga et al. [43] found that endogenous HIF-1α is detectable in nasopharyngeal carcinoma cell-derived exosomes which retains DNA-binding activity and is transcriptionally active in recipient cells after exosome uptake. Exosome-mediated delivery of active forms of HIF-1α results in reciprocal changes in the expression of E-cadherin and N-cadherin associated with EMT and invasiveness [43]. This is of significant interest, since HIF-1α is ubiquitinated by Von Hippel Lindau E3 ligase for proteasome degradation in cytoplasm under normoxic condition [51]. Their results indicate that HIF-1α may keep it safe in exosomes without ubiquitination by Von Hippel Lindau E3 ligase and that exosomal HIF-1α may be internalized by recipient cells and be translocated to nucleus where transcription factors are supposed to function in.

Several proteins with ECM degradation activity, such as MMP-13 [45], MMP14, and C4.4A [49] have been found in exosomes and been demonstrated to involve in tumor migration and invasiveness. It was showed that hypoxic cancer cells have significantly increased expression of MMP13 in both cellular and exosomal milieu [45]. Exosomal MMP-13 significantly up-regulated Vimentin expression while decreasing E-cadherin levels in recipient cells and contribute to cell invasion in vitro and in vivo [45]. Metastasis-associated C4.4A, being frequently associated with hypoxia, is largely unknown about its function. Ngora et al. [49] found that hypoxia promoted the release of C4.4A in exosomes in a HIF-1α independent manner. They further demonstrated that hypoxia-induced recruitment of α6β4 integrin toward exosomal C4.4A, MMP-14 membrane type 1 matrix metalloproteinase/MT1-MMP), and TACE (tumor necrosis factor-α-converting enzyme) allows for a shift from adhesion to motility of recipient tumor cells [49]. These results suggest that hypoxic cancer cells could drive non-hypoxic cells toward pro-metastatic phenotype through exosomes that deliver increased mount of ECM degradative proteinases.

Additionally, adhesion molecules [50] and soluble growth factors/cytokines [43] may also be efficiently involved in exosome-mediated recipient cell migration and invasion. The L1 adhesion molecule (CD171) is increased in exosomes under hypoxic condition. Exosomal CD171 from ascites from ovarian carcinoma patients is a potent inducer of cell migration and can trigger extracellular signal-regulated kinase phosphorylation [50]. Soluble factors, such as TGF-β, TNF-α, IL-6, and IL-10, are enriched in hypoxic exosomes and are essential for the regulation of recipient cell migration [47, 48]. It has also been revealed that proteins that are involved in exosome biogenesis are required for exosome-mediated cancer progression under hypoxia. The small GTPase, RAB22A for example, is induced by hypoxia dependently on HIF-1α and plays an important role in hypoxic exosome-induced focal adhesion, invasion, and metastasis of recipient cells [32].

### Non-coding RNAs (ncRNAs)

Another set of exosomal cargos that play key role in hypoxia regulation is ncRNA, miRNAs and lncRNAs especially. We demonstrated in OSCC that hypoxia increases miR-21 levels in TDEs which could be internalized by normoxic cells and elicit recipient cells toward a prometastatic phenotype through regulating the process of epithelial-mesenchymal transition [31]. In addition, lncRNA-UCA1-rich TDEs derived from bladder cancer cells could promote tumor growth and progression though affecting epithelial-mesenchymal transition of recipient bladder cancer cells [46]. However, the molecular mechanism by which exosomal lncRNAs regulate hypoxic adaption remains largely uncovered. This is not surprising, since many genomic regions transcribed into lncRNAs indicated by ENCODE project are supposed to have distinct biological functions, while only a minority of which having been clarified [52].

Other than invasiveness and metastasis, cell proliferation [46], viability [53], and stemness [48] under hypoxic condition have also been suggested to be influenced by TDEs. Hypoxia-induced exosomal lncRNA-UCA1 not only regulates recipient cell invasion, but also contributes to the proliferation of recipient bladder cancer cells [46]. In hepatocellular cancer, exosomal linc-RoR has been suggested to regulate cancer cell viability under hypoxia through a miR-145-HIF-1α signaling module [53]. Hypoxic TDEs had increased number of proteins (160 proteins) than normoxic TDEs (62 proteins), primarily associated with enhanced stemness of prostate cancer cells under hypoxic TME [48]. However, which protein in TDEs is indeed playing the key role in this process has not been elucidated yet. Besides, the mechanisms by which exosomal lncRNA-UCA1 and linc-RoR regulate proliferation and viability respectively remains insufficiently investigated. Hence, though TDEs with protein and ncRNA cargos have been revealed their roles in regulating cell invasiveness, proliferation, viability, and stemness within hypoxic microenvironment, the mechanisms underlying these regulations need further investigation.

### TDEs regulate stroma cell biology

#### TDEs regulate endothelial cell biology

Angiogenesis, the growth of new blood vessels from pre-existing ones, is an important process in the cancer development mediated mainly by endothelial cells [21]. It has been widely accepted that hypoxia is a certain driving force of tumor angiogenesis through multiple growth factors and signaling pathways [4]. Recently, TDEs are reported to be enriched in pro-angiogenic proteins which stimulated tubule formation by endothelial cells, not even under hypoxic condition [54]. Given that hypoxia could remarkably rebuild exosomal contents, hypoxic TDE-regulated angiogenesis has gained considerable interest and increasingly evidence has been added to the theoretical framework of hypoxia-induced angiogenesis in the last several years. The potential roles of tumor-derived exosomes in angiogenesis have been recently reviewed by Ludwig et al. [55]. Here, we aim to focus on the hypoxia-related protein and nuclear acid cargos within TDEs that are involved in the regulation of tumor angiogenesis.

#### Proteins

Early in 2010, quantitative proteomics performed by Park et al. [56] revealed that hypoxia induced secretion of pro-angiogenic proteins which, unexpectedly, were predominantly cytoplasmic and membrane proteins rather than soluble. By means of ultracentrifugation, 54% of theses secreted proteins were enriched and many of them were exosome-associated such as CD9 tetraspanins and Alix [56]. Though no functional experiment was performed, this proteomic study provided novel evidence to support the notion that proteins in hypoxic TDEs may potentially regulate angiogenesis. Mattias Beltinga and colleagues showed that microvesicles derived from hypoxic glioblastoma (GBM) cells carry tissue factor and factor VIIa with the potential to activate protease-activated receptor 2, which was found to elicit an angiogenic phenotype of endothelial cells [57]. The same group further demonstrated that exosomes derived from hypoxic GBM cells substantially induced microvascular sprouting compared with normoxic cells [42]. Moreover, exosomes isolated from the plasma of GBM patients in all cases significantly stimulated endothelial cell proliferation and survival [42]. Interestingly, endothelial cells were programmed by GBM cell-derived hypoxic exosomes to secrete several potent growth factors and cytokines and to stimulate pericyte PI3K/AKT signaling activation and migration [42].

Recently, Carbonic anhydrase 9, a validated HIF-1α target, was identified in renal cell carcinoma cell-derived exosomes and was increased upon hypoxia treatment, which promoted migration and tube formation of human umbilical vein endothelial cells (HUVECs) [58]. These results suggest that hypoxia-induced pro-angiogenic protein may be wrapped in TDEs which, upon infusion, could remold endothelial cell activity and stimulate angiogenesis.

#### ncRNAs-miRNA

High throughput techniques (i.e. microarrays and next generation sequencings (NGS) have revealed that miRNAs [59] can be regulated by hypoxia in a variety of cell types. Exosomal miRNAs profiles indeed reflect the changes of parent cells to a certain degree. Using a Taq-Man low-density miRNA array, Tadokoro et al. [60] identified a subset of miRNAs, including miR-210, were significantly increased in exosomes secreted from hypoxic leukemia K562 cells. The increased miR-210 in hypoxic cell-derived exosomes was transferred into HUVECs, which then induced the tubulogenesis of HUVECs under normoxic conditions through targeting Ephrin-A3 [60]. Additionally, hypoxic multiple myeloma (MM) cells are found to produce more exosomes with increased miR-135b levels than the parental cells under normoxia conditions. Exosomal miR-135b directly inhibited its target factor–inhibiting hypoxia-inducible factor 1 (FIH-1) in endothelial cells and thereby enhanced endothelial tube formation [61]. Furthermore, increased miR-494 level in lung cancer cell-derived exosomes was found to target phosphatase and tensin homolog (PTEN) and subsequently activate Akt/eNOS pathway in recipient endothelial cells, resulting in an enhancement of angiogenesis [62]. More recently, hypoxic lung cancer-secreted exosomal miR-23a was found to induce angiogenesis through targeting prolyl hydroxylase 1 and 2, leading to the accumulation of HIF-1α in endothelial cells; and targeting tight junction protein ZO-1, bringing about increased vascular permeability and cancer trans-endothelial migration [63]. These results suggest that hypoxia-related exosomal miRNA could influence the biology of endothelial cells and induce angiogenesis in several types of cancer through different regulation pathways.

#### TDE regulate myeloid lineage biology

Cells of myeloid lineage, including monocytes and neutrophils, macrophages, and DCs, represent a group of the most prominent stromal cells in TME [64]. This heterogeneous cell collective plays important roles in tumor immunity, invasion, and progression. Studies by us and several other researchers have showed that hypoxia could redirect the differentiation, induce mobilization and infiltration, and regulate activity of myeloid cells through secretomes by tumor cells, such as growth factors, cytokines, and chemokines [65, 66]. Nowadays, TDEs have made their ways to participate in the regulation of myeloid cell differentiation and function.

### Macrophages

The infiltration of tumor-associated macrophages (TAMs) in the TME is correlated with tumor development. Recently, it was reported that hypoxic pancreatic cancer cells could activate macrophages to the M2 phenotype through delivering miR-301a-3p which activates the PTEN/PI3Kγ signaling pathway in recipient macrophages [67]. Consistently, Chen et al. [68] found that hypoxia induces the expression of miR-940 in exosomes derived from epithelial ovarian cancer, which stimulated M2 phenotype polarization. The same group further demonstrated that exosomes induced by hypoxia, in comparison with normoxic exosomes, express higher levels of miR-21-3p, miR-125b-5p, and miR-181d-5p, which induce M2 macrophage polarization [69]. The M2 polarization of macrophages induced by hypoxic TDEs could facilitate the migration, invasion, and proliferation of tumor cells in return [67–69]. These results suggest that hypoxic pressure on tumor cell alters the miRNA profiles of TDEs, which could regulate the macrophage differentiation and function on tumor cells in a feedback loop.

### Myeloid-derived suppressor cells (MDSCs)

MDSCs represent a heterogeneous population of immature myeloid cells including immature precursors of DCs, granulocytes, and macrophages [70]. MDSCs are expanded during cancer development and have emerged as critical elements of cancer-induced immune dysfunction, playing multiple roles in tumor progression by promoting tumor cell survival, angiogenesis, invasion, and metastasis [70, 71].

Chalmin et al. [72] demonstrated that mouse TDEs mediated an interaction between tumor cells and MDSCs, which determines the suppressive activity of the MDSC via heat shock protein 72 (Hsp72) triggered Stat3 activation. In the meantime, Xiang et al. [73] showed that exosomes released from in vitro cultured B16 tumor cells are capable of inducing MDSC activation and expansion in a Toll-like receptor 2 (TLR2)-dependent manner. Although discrepancies exist between studies, these results actually suggest that TDEs play important roles in restraining tumor immune surveillance by promoting MDSC suppressive functions [72, 73]. Interestingly, both Hsp72 [74] and TLR2 [75] expression are found to be directly stimulated by hypoxia, indirectly suggesting that hypoxic exosome may regulate the MDSCs function. Direct evidence came through the finding that hypoxia-inducible expression of miR-10a and miR-21 in glioma TDEs mediated TDE-induced MDSC expansion and activation by targeting RAR-related orphan receptor alpha (RORA) and PTEN [71]. These results suggest that hypoxia-induced proteins and miRNAs carried by TDEs may remodel MDSC differentiation and function, which influences tumor progress backward.

### TDE regulate lymphocyte biology

#### T cells

Yin et al. [76] observed that microvesicles derived from mouse sarcoma S-180 cells and Lewis lung carcinoma cells could efficiently transport miR-214 to CD4+ T cells, resulting in a downregulation of PTEN and expansion of Tregs. Although they did not mention the contribution of hypoxia on this regulation, miR-214 is indeed induced by environmental hypoxia [77], suggesting that hypoxia may participate in this regulation process. Another miRNA that can be shuttled to T cells by nasopharyngeal carcinoma cell-derived exosomes is miR-24-3p, which inhibits T cell proliferation and T_H_1 and T_H_17 differentiation and induces Treg expansion through targeting fibroblast growth factor 11 in recipient T cells [78]. This process was found to be enhanced under hypoxia [78]. Exosomal protein cargo, TGF-β was found to be delivered to T cells by breast cancer cell-derived exosomes, which is supposed to mediate the hypoxia-induced loss-of-function of recipient T cells [79]. Other than internalization approach described above, Muller et al. [80] demonstrated that exosomes derived from head and neck squamous cell carcinoma (HNSCC) cells and plasma of patients with HNSCC or acute myelogenous leukemia induced Ca2+ influx in recipient T cells by means of surface contact. These results suggest that hypoxia may influence the function of T cells in TME by miRNAs and proteins delivered by TDEs.

#### NK cells

TDEs have been demonstrated to have either stimulatory or inhibitory roles on anti-tumor immune response by NK cells. For instance, TEDs derived from hepatocellular [81], pancreatic [82], and colon [83] carcinoma cells are able to enhance the cytotoxicity of NK cells through transferring proteins such as HSP and HLA-B-associated transcript 3. However, this stimulation may probably be dysregulated under hypoxic microenvironment. Hypoxia induced a remarkable increase of TGF-β in exosomes derived from IGR-Heu (a lung carcinoma cell line) and K562 (a chronic myelogenous leukemia cell line) cells, which inhibited cytotoxicity and IFN-γ production by NK cells through NKG2D inhibition [84]. Additionally, hypoxia-induced miR-23a in TDEs operated as an additional immunosuppressive factor of NK cells through directly targeting CD107a which is an established marker of NK cell functional activity [84]. These results highlight the role of hypoxia-regulated TDEs on immunodeficiency within TME.

#### TDEs regulate fibroblast biology

Fibroblasts are one of the most abundant cell types in the cancer stroma. It was showed that exosomes derived from chronic lymphocytic leukemia (CLL) cells could actively induce a shift of endothelial and bone marrow MSCs toward a cancer-associated fibroblast (CAF) phenotype [85]. Ramteke et al. [48] cultured human prostate cancer cells under hypoxic (1% O_2_) or normoxic (21% O_2_) conditions, and exosomes isolated from the conditioned media. They showed that hypoxic cancer cell-derived exosomes, compared with paired normoxic ones, remarkably induced the expression of α-SMA (an established biomarker for CAFs) expression in recipient prostate fibroblasts, suggesting that hypoxia is involved in the regulation of exosome-mediated CAF differentiation.

#### TDEs regulate mesenchymal stromal cells (MSCs) biology

MSCs are a heterogeneous group of progenitor cells with the ability to differentiate into bone, cartilage, adipocytes, fibrocytes, and hematopoietic supporting tissues and are important for tissue regeneration [86]. There are multiple evidences that interactions between tumor cells and MSCs within tumor microenvironment play major roles in supporting cancer progression [87]. Recently, exosomes are found to mediate the crosstalk between tumor cells and MSCs. Paggetti et al. [85] showed that CLL-derived exosomes could shuttle anti-apoptotic proteins, angiogenic factors, and miRNAs (i.e. miR-150 and miR-146a) to MSCs, resulting in a redirection of MSCs differentiation. Intriguingly, HIF-1α was stimulated and activated in MSCs treated by CLL-derived microvesicles, leading to increased production of vascular endothelial growth factor (VEGF) and modified microenvironment in favor of CLL survival and resistance to chemotherapy [88]. Further investigation is warranted for addressing the direct regulation by hypoxia on TDEs-MSCs interaction in TME.

### Stromal cell-derived exosomes in hypoxic microenvironment

**Fig. 2:**
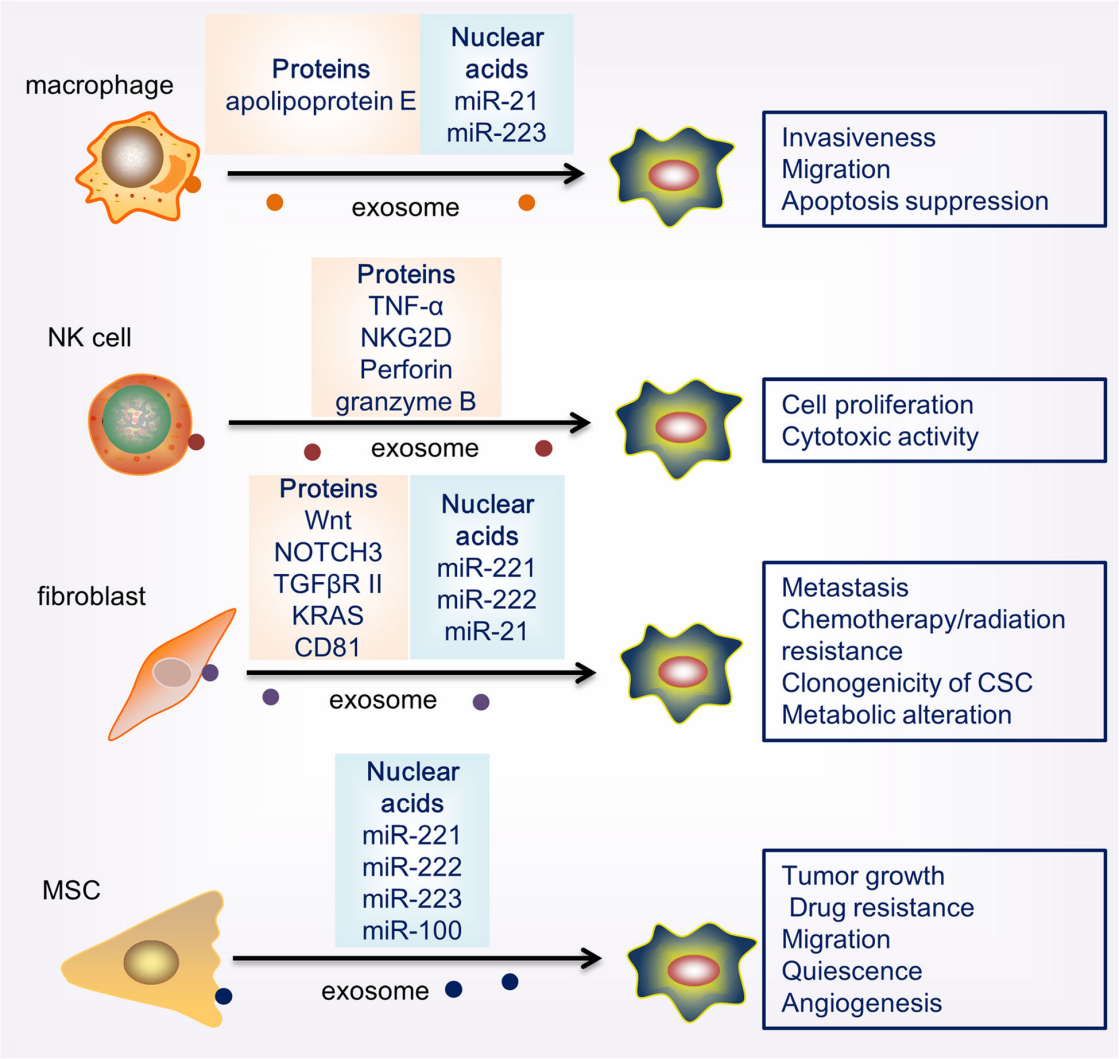
A schematic representation of stromal cell-derived exosomes on cancer cell function in hypoxic TME

Given that exosomes are secreted by nearly all cell types, exosomes derived from stromal cells could, vice versa, have potential impact on cancer cells and other types of stromal cells in the hypoxic microenvironment (Fig. 2).

### Exosomes from stromal cells to tumor cells

#### Macrophage-derived exosomes (MEXs) to tumor cells

Macrophages within the TME facilitate angiogenesis and extracellular-matrix breakdown and remodeling and promote tumor cell motility mainly through releasing angiogenic factors, matrix metalloproteases, growth factors, and cytokines [89].

Proteomic study revealed that MEXs, compared with macrophage cell line-derived exosomes, were mainly enriched in RNA processing, proteasome subunits, and ribosomal proteins, indicating that MEXs possessed higher proteolytic activity [90]. Exosomes released by macrophages could deliver miR-223, a miRNA specific for IL-4-activated macrophages, to co-cultured breast cancer cells, eliciting cancer cell invasiveness through targeting Mef2c-b-catenin pathway [91]. In addition, M2 polarized macrophages were found to promote cisplatin resistance in gastric cancer cells through exosomal delivering of miR-21 which suppresses cell apoptosis and enhances activation of PI3K/AKT signaling pathway by down-regulation of PTEN [92]. Moreover, MEXs have been also found to promote the migration of gastric cancer cells by transfer of functional apolipoprotein E [93]. Intriguingly, miR-223 [94], miR-21 [95], and apolipoprotein E [96] have been reported to be negatively or positively regulated by hypoxia/HIF-1α in several cell types. However, it remains unidentified whether this regulation exists in TAMs within hypoxic TME and whether hypoxia- regulated miR-223, miR-21, and apolipoprotein E could affect the exosome-mediated cancer invasiveness.

#### NK cell-derived exosomes (NK-Exos) to tumor cells

It has been shown that NK-Exos not only express both typical NK markers (i.e., CD56) and killer proteins (i.e., FASL and perforin) but also exert antitumor and immune homeostatic activities [97, 98]. NK-Exos are found to secrete TNF-α which affected the cell proliferation signaling pathway and exert cytotoxic activity against different human tumor target cells [98, 99].

These profiles of NK-Exos warrant them an attractive therapeutic candidate for cancer. In TME, particularly hypoxic milieu, the NK cell activation and cytotoxicity are impaired by multiple mechanisms [100]. Although hypoxia did not alter surface expression of NK cell ligands (HLA-ABC, MICA/B, and ULBP1–2) and receptors (KIR, NKG2A/C, DNAM-1, NCRs and 2B4), it did decrease the expression of the activating NKG2D receptor and intracellular perforin and granzyme B [101]. In fact, the role of NKG2D is now emerging in the control of tumorigenesis, supported by the upregulation of NKG2D ligands in tumor cells [99]. Thus, hypoxia is found to reduce NK cell killing of MM cell in an oxygen dependent manner [101]. However, there is no direct evidence so far linking hypoxia and the cytotoxicity of NK-Exos, which is fundamentally important for NK-Exo-based cancer therapy and needs further investigation.

#### Fibroblast-derived exosomes to tumor cells

Hypoxic cancer cells promote an activation of CAF which, in turn, are able to support angiogenesis as well as cancer cells invasiveness, stemness, chemoresistance, and immune cells recruitment in the TME [102]. Zhao et al. [103] found that CAF-derived exosomes could be taken up by cancer cells in a KRAS-independent manner and are capable of inhibiting mitochondrial oxidative phosphorylation, thereby increasing glycolysis and glutamine-dependent reductive carboxylation in cancer cells. Interestingly, reductive carboxylation is a critical pathway to support the growth of tumor cells under hypoxia. These results, indeed, suggest that CAF-derived exosomes could induce metabolic alterations in cancer cells via mimicking hypoxia- induced environment [103]. Functional studies performed by Au Yeung et al. [104] showed that miR-21 is transferred from CAFs to the cancer cells, where it suppresses ovarian cancer apoptosis and confers chemoresistance by binding to its direct novel target, APAF1.

Since miR-21 is increased under hypoxia in a HIF-1α dependent manner [95], this results suggest an potential involvement of hypoxia in CAF-derived exosome-mediated cancer progression. Thus far, there still lack direct evidence to demonstrate whether hypoxia could modify the profile and function of CAF-derived exosomes in TME. Proteomic analysis performed on cardiac fibroblasts revealed that hypoxia selectively increase expression of proteins with ECM and signaling annotations in cardiac fibroblast-derived exosomes [105]. This could be an indication that hypoxic TME may be able to modify the CAF-derived exosomal cargo and function, which needs further investigation though.

#### MSC-derived exosomes (MSC-Exos) to tumor cells

MSCs from gastric cancer tissues could favor cancer growth and migration by transferring exosomal miR-221 to cancer cells [106]. Additionally, MM-derived MSCs secret exosomes, with a lower content of the tumor suppressor miR-15a and higher levels of oncogenic proteins, cytokines, and adhesion molecules, could promote MM tumor growth [107]. Moreover, Bliss et al. [108] reported that breast cancer cells prime MSCs to release exosomes containing miR-222/223, which in turn promotes quiescence in a subset of cancer cells and confers drug resistance. They found that systemic administration of MSC loaded with antagomiR-222/223 sensitized breast cancer cells to carboplatin-based therapy and increased host survival [108]. Recently, it was showed that MSC-derived exosomes with miR-100 cargo could modulate mTOR/HIF-1α signaling axis in recipient breast cancer cells, resulting in a decrease in VEGF expression [109]. These results support the notion that exosome-mediated mutual signaling exchange between MSCs and cancer cells mediates cancer progress and hypoxia signaling is involved in. The role of MSC-derived exosomes in stromal remodeling, tumor progression, and cancer immunotherapy has been intensively reviewed by Farah Fatima and Muhammad Nawaz [110]. However, no direct evidence on the role of hypoxia on MSC-derived exosome function has been revealed yet.

Hitherto, there is limited evidence on the direct regulation by hypoxia on stromal cell-derived exosome secretion and content profiles, as well as their function on cancer cell biology. Since exosomes derived from T lymphocytes [20], DCs [111], and NK [97] cells exhibit characteristics and functions from their parent cells, exosomes derived from these cells are now being considered potential candidates for cancer therapy. Whether the distribution and uptake of these exosomes by cancer cell would be modified under hypoxic microenvironment remains largely unknown, which is presumably a critical step for exosome-based therapy. In addition, whether hypoxia could affect stromal cell-derived exosome production as well as content profiles is underinvestigated so far. In the future, it is interesting to know more about the role of hypoxia on stromal cell-derived exosome function, which is a critical branch of the bidirectional signaling transfer by exosomes between cancer cells and stromal cells within hypoxic TME.

### Exosomes from stromal cells to stromal cells

The general involvement of exosomes in intercellular communication suggests that they may contribute to the exchange of biological information within stromal elements, which may mobilize and re-localize the oncogenic factors as well as immune cells that shape the tumor environment [110].

### DC-derived exosomes (DEXs) to stromal cells

Exosomes derived from DCs have been well documented to inherit the antigen-presenting profile from their parent cells. Thus DEXs can modulate recipient cells in a manner beyond classical ligand/receptor signaling pathways, which creates complex cellular modifications that may play substantial roles in tumor development and immune responses [111]. DEXs could be taken up by NK cells, T lymphocytes, and DCs as well and thereby modulate the recipient cell function.

DCs at different stages of maturation release exosomes with different miRNA content [112] as well as MHC I/II class/peptide complexes [11]. These bioactive contents could be delivered to NK cells, T lymphocytes, and DCs as well upon internalization, hemifusion, and fusion with the recipient cells.

It was demonstrated that DEXs express functional IL-15Rα and NKG2D ligands, which promoted recipient NK cell [113] and CD8+ T cell [114] proliferation and activation. However, the regulatory effect of hypoxia on DCs in the TME has not been well clarified yet. Available literature suggests that hypoxia may favor the ability of DCs to induce immune responses, due to the fact that hypoxic DCs have up-regulated expression of pattern recognition receptors (e.g. CD180), components of complement receptor (e.g. Toll-like receptor-1/2 and C-type lectin receptors), and immunoregulatory receptors (e.g. immunoglobulin-Fc receptors) [115]. However, effect of hypoxia on the biogenesis, production, contents, and antigen-presenting functions of DEXs remains underinvestigated, which might be an attractive topic in the future.

### Macrophage-derived exosomes to stromal cells

We mentioned above that exosomes released by macrophages could deliver miR-223 to co-cultured cancer cells to elicit an invasive phenotype of breast cancer [91]. These miR-223 containing MEXs could also be transported to stromal cells, including monocytes, endothelial cells, epithelial cells, and fibroblasts [116]. miR-223 delivered by exosomes derived from activated macrophages induce the differentiation of recruited monocytes toward macrophages [116]. Recently Cheng et al. demonstrated that exosomes derived from M1-polarized, proinflammatory macrophages displayed a tropism toward lymph nodes after subcutaneous injection, primarily taken up by the local macrophages and DCs, and they induced the release of a pool of Th1 cytokines [117]. Interestingly, M1, but not M2, exosomes induced a stronger antigen-specific cytotoxic T cell response [117]. Though, they did not dig into the molecular mechanisms underlying the MEX-induced vaccination and T cell response. Hypoxia may potentially participate in the MEX-regulated macrophage differentiation as well as T cell response, because elevated HIF-1α has been shown to trigger a decrease of miR-223 in pulmonary artery smooth muscle cells [94]. If this HIF-1α-dependent downregulation of miR-223 exists in macrophages, MEX-regulated macrophage differentiation and T cell response may be negatively influenced in hypoxic TME.

### MDSC-derived exosome (MDSC-Exo) to stromal cells

Mass spectrometry and RNA-sequencing showed that MDSC-Exos carry proteins, mRNAs, and microRNAs, some of which have known or predicted functions consistent with MDSC suppressive activity [118]. Functional study showed that MDSC-Exos are taken up by T cells, macrophages, and NK cells, resulting in a strong increase in Treg, reduced T helper proliferation, mitigated cytotoxic activity, and slight increase in lymphocyte apoptosis [119]. However, in this study, the molecular mechanism underlying the MDSC-Exo-mediated immunosuppression was not investigated. Deng et al. [120] found that doxorubicin-treated 4 T1 breast tumor bearing mice had an increase of miR-126 + MDSCs which produce miR-126a-rich exosomes. The miR-126-rich MDSC-Exos, upon transferred to T cells, significantly suppressed Th1 cell proliferation and IFN-γ secretion and induced Th2 cell responses. These effects finally contributed to tumor angiogenesis, lung metastasis, and chemo resistance [120]. We and other researchers have shown that hypoxia, mainly through HIF-1α, indeed regulate MDSC recruitment [65], differentiation [121], and immunosuppressive function [122]. However, no study yet, to the best of our knowledge, has reported the direct role of hypoxia on MDSC-Exo production, exosomal content profile, or immunoregulatory function. Since miR-126 was found to inhibit HIF-1α protein expression and miR-126 deactivation induced a pseudohypoxia in renal cell carcinoma model [123], it indicates a potential correlation between hypoxia and miR-126+ MDSC-Exo-mediated immunosuppression.

## Conclusions

Cancer cells and stromal cells within TME are influenced by the physical and chemical milieu, and crosstalk between cells may also dynamically reshape the microenvironment in a feedback manner. Exosomes, science being discovered as a signaling carrier and communication media, have waved themselves the forefront of cancer research in the last decade. In hypoxic microenvironment, the production of exosomes as well as exosomal protein and nuclear acid profiles may be influenced by the shortage of oxygen and the acid circumstance, which makes exosome a potential noninvasive approach for diagnosis of tumors with hypoxia. Indeed, the application of exosomes as a minimally-invasive circulating biomarker platform has gained much attention and is under intensive investigation.

Exosomes mediate a broad range of bidirectional signal transductions between a variety of cell types (cancer cell-cancer cell, cancer cell-stromal cell, and stromal cell-stromal cell) within hypoxic TME, playing essential roles in tumor invasiveness, angiogenesis, proliferation, chemotherapy and radiation resistance, immune evasion, metabolism, and cancer stemness. Exosomes derived from tumor cells have been widely invested for their roles in regulating the biology of recipient tumor cells and diverse stromal cells in hypoxic TME. The stromal cell-derived exosomes, however, remains underinvestigated for their roles in the regulation of hypoxic TME, which is undeniably significant in the dynamics of hypoxic TME and cancer progression. Given that exosomes derived from T cells, DCs, and NK cells exhibit both anti-tumor effects and immunoregulatory functions, the potential use of exosome as an immunotherapy reagent or as a drug delivery platform has obtained considerable scientific interest. However, the production, infiltration, distribution, and incorporation of the stromal cell-derived exosomes under hypoxic conditions are of significant importance for exosome-based therapeutics and far from well elucidated yet. A more comprehensive understanding of exosomes and their molecular cargos, stromal origin especially, in the regulation of hypoxic TME is challenging and fascinating for the improvement of detection and treatment of hypoxic tumors in the future.

## Abbreviations

CAF: Cancer-associated fibroblast
CLL: Lymphocytic leukemia
DC: Dendritic cell
DEX: DC-derived exosome
ECM: Extracellular matrix
EMT: Epithelial to mesenchymal transition
ENCODE: Encyclopedia of DNA Elements
EV: Extracellular vesicle
FASL: Fas ligand
FIH-1: Factor–inhibiting hypoxia-inducible factor 1
GBM: Glioblastoma
HIF: Hypoxia-inducible factor
HLA: Human leukocyte antigen
HNSCC: Head and neck squamous cell carcinoma
Hsp72: Heat shock protein 72
HUVEC: Human umbilical vein endothelial cells
KIR: Killer-cell immunoglobulin-like receptor
MDSC: Myeloid-derived suppressor cell
MDSC-Exo: MDSC-derived exosome
MEX: Macrophage-derived exosome
MHC: Major histocompatibility complex
MICA: MHC class I polypeptide-related sequence A
miRNA: microRNA
MM: Multiple myeloma
MMP: Matrix metalloproteinase
MSC: Mesenchymal stromal cell
MSC-Exo: MSC-derived exosome
NCR: Natural cytotoxicity receptor
ncRNA: Non-coding RNA
NGS: Next generation sequencings
NK: Natural killer cell
NK-Exo: NK cell-derived exosome
OSCC: Oral squamous cell carcinoma
PDGF: Platelet-derived growth factor
PRAS40: Proline-rich Akt substrate of 40 kDa
PTEN: Phosphatase and tensin homolog
ROCK: RHO-associated protein kinase
RORA: RAR-related orphan receptor alpha
TACE: Tumor necrosis factor-α-converting enzyme
TAM: Tumor-associated macrophage
TDE: Tumor derived exosome
TGF-β: Transforming growth factor beta
TLR2: Toll-like receptor 2
TME: Tumor microenvironment
TNF-α: Tumor necrosis factor alpha
Treg: Regulatory T cell
ULBP1: UL16 binding protein 1
VEGF: Vascular endothelial growth factor
